# Adolescents’ perceived barriers and facilitators to seeking and accessing professional help for anxiety and depressive disorders: a qualitative interview study

**DOI:** 10.1007/s00787-020-01707-0

**Published:** 2021-01-27

**Authors:** Jerica Radez, Tessa Reardon, Cathy Creswell, Faith Orchard, Polly Waite

**Affiliations:** 1 School of Psychology and Clinical Language Sciences, University of Reading, Reading, RG6 6AL UK; 2 The Oxford Institute of Clinical Psychology Training and Research, University of Oxford, Oxford, OX3 7JX UK; 3 Departments of Experimental Psychology and Psychiatry, University of Oxford, Oxford, OX2 6GG UK; 4 School of Psychology, University of Sussex, Brighton, BN1 9RH UK

**Keywords:** Adolescence, Anxiety disorders, Depressive disorders, Help-seeking, Access, Barriers

## Abstract

Anxiety and depressive disorders are the most common mental health disorders in adolescents, yet only a minority of young people with these disorders access professional help. This study aims to address this treatment gap by improving our understanding of barriers and facilitators to seeking/accessing professional help as perceived by adolescents with anxiety/depressive disorders identified in the community. Twenty-two adolescents, aged 11–17 years, who met diagnostic criteria for a current anxiety and/or depressive disorder were identified through school-based screening. In-depth qualitative interviews were conducted one-to-one with each adolescent and adolescents’ parents were interviewed separately for the purpose of data triangulation. Data were analysed using reflexive thematic analysis. We identified four themes capturing adolescent perceived barriers and facilitators to seeking/accessing professional help for anxiety and depressive disorders: (1) making sense of difficulties, (2) problem disclosure, (3) ambivalence to seeking help, and (4) the instrumental role of others. Barriers/facilitators identified within each theme reflect important developmental characteristics of adolescence, such as a growing need for autonomy and concerns around negative social evaluation. At the same time, the results highlight adolescents’ dependency on other people, mainly their parents and school staff, when it comes to successfully accessing professional help for their mental health difficulties. This study identifies a number of barriers/facilitators that influence help-seeking behaviour of adolescents with anxiety and/or depressive disorders. These factors need to be addressed when targeting treatment utilisation rates in this particular group of young people.

**Supplementary Information:**

The online version contains supplementary material available at 10.1007/s00787-020-01707-0.

## Introduction

Anxiety and depressive disorders are the most common mental health disorders in adolescence, with estimated prevalence rates of 5% (depressive disorders) and 8% (anxiety disorders) [1–4], and they commonly co-occur in adolescents [5]. However, only two-thirds of adolescents with anxiety or depressive disorders seek and access any professional help, and only a minority access specialist mental health support [2, 3]. Understanding the barriers to seeking/accessing help is crucial to address this treatment gap.

Reasons underlying low treatment rates for anxiety and depressive disorders in adolescents are complex. Limited service provision and long waiting times represent a significant logistical barrier to accessing specialist mental health [2, 6, 7]. A lack of mental health knowledge, including difficulties with mental health problem identification, negative views, and attitudes towards mental health and help-seeking, and family circumstances can also stop adolescents and their families from seeking and accessing help for mental health problems [8–10].

In addition, a recent systematic review of young people’s perceived barriers and facilitators to seeking and accessing professional help for their own mental health problems [8] identified perceived societal views and negative attitudes towards mental health and help-seeking (e.g., stigma and embarrassment), and perceiving help-seeking as a sign of one’s weakness as the most frequently reported barriers. Factors that facilitated young people in help-seeking were positive attitudes and encouragement from their support network and positive perceptions of the contact between them and professionals when seeking/accessing help. However, studies in this review were highly heterogeneous and particular barriers and facilitators for adolescents with specific mental health problems (such as anxiety or depressive disorders) were not investigated. Furthermore, many studies have explored views about barriers and facilitators either exclusively among young people who have successfully accessed a specialist mental health services [11, 12], or among the general population [13, 14] (many of whom may not have experienced mental health difficulties or ever needed to access professional help or services). This means that the experiences of those who meet the diagnostic criteria for specific mental health problems but have not necessarily reached a specialist mental health service have not yet been captured, including those who have not sought any professional help, and those who may have sought help through their school or GP but not accessed specialist services. Finally, given the wide age range of participants across studies, the particular barriers and facilitators faced by adolescents remain unclear. This is important as adolescents both differ in their clinical characteristics to children [15] and can take a more active role in help-seeking/accessing [16]. Similarly, existing help-seeking models for young people, such as the model of help-seeking developed by Rickwood et al. [17], do not consider age and disorder-specific barriers and facilitators. Together, these limitations of the extant literature highlight the need for a detailed understanding of what helps and hinders help-seeking and accessing in specific groups of young people.

This study aimed to address gaps in the existing literature by improving understanding of how young people with a diagnosis of an anxiety and/or depressive disorder, identified in a community setting (i.e., not through mental health services), perceive seeking/accessing professional help. The study addressed the limitations of previous studies with community samples [8] using ‘gold standard’ diagnostic interviews to identify participants. The study aimed to identify adolescents’ perceived barriers and facilitators to seeking/accessing professional help. Given that the process of seeking help for adolescents is complex and not yet fully understood, a qualitative approach was chosen to explore this from the perspectives of young people.

## Method

The study was approved by the University of Reading Research Ethics Committee (UREC 18/28). We used the techniques suggested by Mays and Pope [18] to ensure the quality and rigour of the study, and followed the COREQ checklist (see Online Resource 1) for explicit and comprehensive reporting of qualitative studies [19].

### Recruitment and participants

**Fig. 1:**
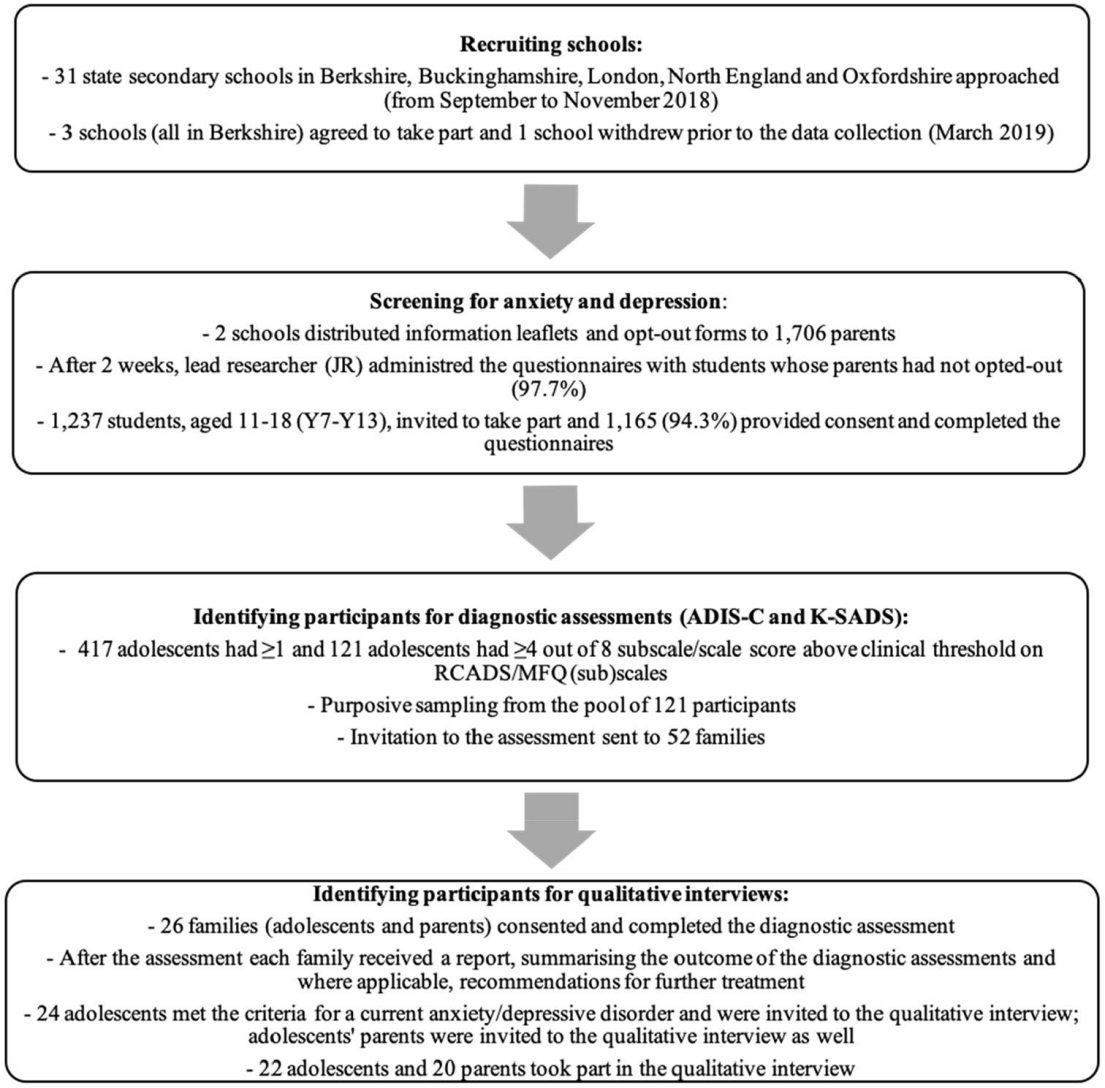
The process of recruiting participants for the current study

Participants were recruited through two large mixed state secondary schools in Berkshire, UK, as part of a wider study, including whole school screening for anxiety and depressive disorders (Radez et al. under review). The process of recruitment for the current study is outlined in Fig. 1, and described in more detail in Online Resource 2.

Of 26 adolescents (aged 11–18) who took part in the diagnostic assessment, 24 met diagnostic criteria for an anxiety and/or depressive disorder. These adolescents and their parents/carers were invited to take part in qualitative interviews. Although the primary focus of the study was adolescents’ perceived barriers/facilitators, their parents were also invited to take part in a separate qualitative interview for the purpose of data triangulation. Each participant (adolescent and parent) provided written consent to take part in the interview and to allow the researcher to audio record the interview. If the young person was under 16 years, they provided written assent and their parent provided written consent. In total, 22 adolescents and 20 of their parents took part in the qualitative interviews. The lead researcher (JR) conducted all interviews with adolescents and parents separately, and all interviews were conducted within one session. During qualitative interviews adolescents and parents also reported other diagnoses (e.g., autism spectrum disorder, gender dysphoria, and physical conditions), which have not been assessed during the diagnostic assessment. Adolescents were interviewed one-to-one in a quiet, private room in their school, and parents were interviewed over the phone at a time that was convenient for them. In 18 cases, parent interviews were conducted with adolescents’ mothers and in two cases with adolescents’ fathers. Two parents/carers did not take part without giving any reason. Each family that took part in the qualitative interview was given £10 voucher to reimburse them for their time. Adolescents’ demographic and clinical characteristics are outlined in Table 1.

**Table 1:** Adolescents’ demographic and clinical characteristics

Pseudonym	Age	Gender	Accessed professional help for anx/dep in last 12 months^a^	Primary diagnosis (CSR)	Secondary diagnoses (CSRs)
Luke	11	Male	Yes	SpecP (spiders) (5)	GAD (4), SepA (4), SocA (4)
Savannah	11	Female	Yes	SocA (5)	GAD (4)
Claire	12	Female	Yes	MDD (6)	GAD (4)
Ben	12	Male	No	MDD (5)	GAD (4)
Zara	12	Female	No	GAD (5)	SocA (4)
Tim	12	Male	Yes	GAD (5)	
Harry	12	Male	No	MDD (5)	SocA (5), GAD (5)
Debbie	12	Female	Yes	GAD (6)	SepA (5), SocA (4)
Katie	13	Female	No	PD (7)	Agor, GAD, SocA
Maya	13	Female	Yes	Agor (5)	SocA (5), GAD (4), Hallucinations and delusions
Isaac	13	Male	No	MDD (6)	PD (6), SocA (5), GAD (4), SpecP(darkness) (4)
Frank	14	Male	No	MDD (6)	GAD (5), SocA (4)
Diane	14	Female	Yes	GAD (6)	SocA (5)
Victoria	14	Female	No	SocA (4)	
Hannah	14	Female	No	PD (5)	SocA (5), Agor (4), GAD (4)
Lilly	15	Female	Yes	GAD (6)	SocA (6), PD (6), Agor (5), MDD (5), Dyst (5), SepA (4)
Chris	15	Male	No	PD (7)	GAD (5), Agor (5), SocA (5)
Alex	15	Male	Yes	GAD (6)	SocA (5)
Anna	15	Female	No	GAD (6)	PTSD (7), SocA (5), Dyst (5)
Tina	15	Female	Yes	GAD (6)	SocA (4), Dyst (5)
Sophie	16	Female	No	GAD (6)	SocA (5), Dyst (5), Agor (4)
Joe	16	Male	Yes	SocA (5)	GAD (4)

*ADHD *attention deficit hyperactivity disorder, *Agor *agoraphobia, *anx *anxiety disorder, *ASD *autism spectrum disorder, *dep *depressive disorder, *Dyst *dysthymia, *GAD *generalised anxiety disorder, *MDD *major depressive disorder, *PD *panic disorder, *SocA *social anxiety disorder, *SpecP *specific phobia

^a^Adolescent received professional support within school or outside the school (e.g., specialist mental health services, counselling) for anxiety and/or depression in last 12 months

Of the 22 adolescents, 16 (72.7%) identified as White-British and 6 (27.3%) as other varied ethnic groups. Seven (31.8%) adolescents and/or their parents also reported that a young person had additional physical or mental health difficulties (e.g., chronic physical illness, autism spectrum disorder, attention deficit hyperactivity disorder, dyslexia, Tourette syndrome, and gender dysphoria) that had been diagnosed by other professionals.

### Measures

#### Questionnaire measures

*Revised Child Anxiety and Depression Scale, Child Version—RCADS-C* [20]. The RCADS-C is a 47-item self-report questionnaire measure of symptoms of anxiety and low mood in young people, aged from 8 to 18. The questionnaire consists of six subscales that correspond to DSM-IV anxiety/depressive disorders—separation anxiety disorder (SAD), social phobia (SP), obsessive–compulsive disorder (OCD), panic disorder (PD), generalised anxiety disorder (GAD), and major depressive disorder (MDD). Respondents rate how often each item applies to them, using a four-level scale from 0 (‘never’) to 3 (‘always’). The RCADS demonstrates favourable psychometric characteristics when applied in various settings (e.g., clinic and community) and in different countries [21]. In the current study, subscale scores and anxiety/depression/total scores and standardised *T *scores were calculated using syntax provided on the author’s website. A *T* score of > 70 indicated a clinically significant level of anxiety/depression symptoms. Adolescents’ scores on anxiety total scale and on six subscales were used to identify participants for the diagnostic assessment.

*Moods and Feelings Questionnaire, Child Version MFQ-C* [22]. The MFQ-C is a 33-item self-report screening tool for depression in children and young people, aged between 6 and 17. Respondents are asked to report how they have been feeling or acting in the past 2 weeks. For each item, they can respond with ‘not true’ (0), ‘sometimes’ (1), or ‘true’ (2). Research studies suggest that the MFQ provides reliable and valid measure of depression in children and young people in both clinical and community samples [23]. In the current study, the MFQ-C total score was calculated by summarising participants’ responses to all 33 items. Based on previous research [24], we used the cut-off score > 26 to identify adolescents with a clinically significant level of depressive symptoms.

*Help-seeking questions* Each adolescent was asked three questions about seeking/accessing professional help in the last 12 months. Adolescents reported whether they (1) had spoken to a professional (e.g., teacher or GP) about feeling anxious/depressed in the last 12 months, (2) had received any support from a professional to help them with difficulties with anxiety/depression in the last 12 months, and (3) felt that they would benefit from professional support for anxiety/depression. Adolescents’ responses to these three questions were used to purposively sample participants for diagnostic assessments.

#### Diagnostic interviews

The following diagnostic assessments were administered to identify participants who met diagnostic criteria for an anxiety and/or depressive disorder and were therefore eligible for the qualitative study. All interviews were administered by the first author (JR), trained to reliably deliver the diagnostic assessments. Following assessment, each case was discussed in diagnostic supervision with co-author (FO), who has extensive experience of delivering, training, and supervising these diagnostic tools. Agreement between JR and FO was excellent [presence/absence of diagnoses, *κ* = 0.820, Clinical Severity Rating (CSR) rating, ICC = 0.956].

*Anxiety Disorder Interview Schedule—Child Version—ADIS-IV-C* [25]. The ADIS-IV-C is a standardised diagnostic interview, based on the DSM-IV-TR designed to assess anxiety and other disorders in children and adolescents. In the present study, the anxiety sections of the ADIS-IV-C were used to determine whether the adolescent met diagnostic criteria for any anxiety disorder. Minor adaptations to the interview schedule were made, so the diagnoses were assigned based on the DSM-5. If the adolescent met symptom criteria for a diagnosis, then the assessor would assign a Clinician Severity Rating (CSR), ranging from 0 to 8; a CSR of 4 or more would indicate that the young person met criteria for diagnosis. The diagnosis with the highest CSR was considered as the primary diagnosis. Studies using the ADIS-IV-C provide strong evidence for its good psychometric characteristics, which has been especially the case for the anxiety section [26]. Furthermore, ADIS-C provides reliable and valid information even when administered with child only, and reliability of child report is especially high for older children/adolescents [27].

*Kiddie Schedule for Affective Disorders and Schizophrenia—Present and Lifetime Version—K-SADS-PL* [28]. The K-SADS-PL is a semi-structured interview for affective disorders and schizophrenia, based on DSM-5. In the present study, the depression and mania sections of the K-SADS-PL child interview were used to determine the presence of depression in adolescents. The diagnosis of the major depressive disorder (MDD) was assigned if a young person met at least five criteria for MDD. In addition, CSR scores were assigned in a similar way as the ADIS-C to provide a comparable estimate of the symptom severity. K-SADS-PL is a diagnostic interview with favourable psychometric characteristics, and is recommended over ADIS in terms of identifying mood disorders in young people [29], with adolescent self-report being particularly informative and reliable [30].

#### Qualitative interviews

The interview topic guides (see Online Resource 3) were developed by the first author (JR), with input from co-authors (TR and PW), drawing on findings of a recent systematic review on barriers/facilitators to seeking professional help for mental health problems in young people [8] and interview guides used in previous similar studies [31]. Areas of inquiry and sample questions for adolescent interview are outlined in Table 2. Although areas of inquiry for parent and adolescent interview were similar, adolescent responses partially guided their parent’s interview. Prior to the data collection, interview questions were piloted with two families (two adolescent girls and their mothers) to help pace the interview and to test the appropriateness of the questions.

**Table 2:** Areas of inquiry and sample questions from adolescent interview topic guide

Area of questioning	Sample questions (probes)
Knowledge and understanding of anxiety and depression in young people	Can you tell me a bit about what you know about anxiety and depression? (Probe: how can you tell if someone your age has been experiencing anxiety and/or depression?)
Personal experience of identifying anxiety and/or depression	Last time we met I asked you lots of questions about how you’ve been feeling recently and you told me about your worries and/or low mood. To what extent do you perceive these feelings to be a problem for you? (Probe: what makes you think that this is (not) a problem?)
Help-seeking attitudes and knowledge about available help/support	Can you tell me a bit about what you know about available help/support for young people experiencing anxiety and/or depression? (Probe: would you know where to find help for experiencing anxiety and/or depression? Where would you go?)
Help-seeking/accessing experience and barriers/facilitators to help-seeking/accessing	Has anything stopped you from seeking help? (Probe: has anything or anyone helped you when trying to seek help?)

The semi-structured interviews were conducted by the first author (JR), a female PhD student in psychology, trained in qualitative research methods and with a background of working in mental health research settings. As JR also conducted the diagnostic assessments with the adolescents and their parents, she had already established a relationship with them; this may have helped them feel more at ease and able to open up, but may also have affected what information they gave in the interview. As English is not the interviewer’s first language, during each interview, she frequently summarised information provided by the participant to ensure that her understanding was accurate [32]. Field notes and initial ideas were written after each interview, and used to partially guide the remaining interviews. All 42 interviews were audio-recorded and transcribed verbatim by JR. Adolescent interviews ranged from 13 to 48 min (M = 28:17, SD = 8:06), and parent interviews from 14 to 77 min (M = 35:36, SD = 14:05).

### Data analysis

Data analysis started, while data collection was ongoing. Data were analysed by the lead researcher (JR), following six phases of the reflexive thematic analysis [33, 34]. We approached the data from an essentialist/realist epistemological orientation, which draws on the experiences, meanings, and the reality of participants. We analysed the dataset inductively (directed by the content of the data) and semantically (reflecting the explicit content of the data). JR familiarised herself with the data by listening to the audio recordings and transcribing the interviews. During transcribing, all identifiable information was removed and participants were given pseudonyms. Adolescents’ interview transcripts were coded following guidance by Saldana [35]. Data were managed and stored using software NVivo, Version 12, QSR International Pty Ltd [36]. Coding was iterative and cyclical, and systematic over all adolescent interview transcripts (i.e., giving full and equal attention to each aspect of the dataset and coding for implicit and explicit contents). Coding was led by JR with regular discussion/input from other team members (TR, PW) with qualitative expertise, to reflect on the coding process. Although all 22 adolescent interviews were coded, additional data did not contribute to new codes after the first 15 transcripts, and therefore, we judged data saturation to have been reached. After all adolescent interview transcripts were coded, JR coded parent interviews using the final set of codes identified in adolescent interviews (i.e., a ‘top–down’ approach). Notably, as parents were interviewed only for the purpose of the data triangulation for the current study, only sections relevant to the research question were coded and analysed. Adolescent and parent interviews were treated as separate datasets, and JR especially looked for elements in the parent dataset that appeared to contradict or was not contained in the adolescent dataset. JR then organised the final set of codes into preliminary themes and subthemes that explained the vast majority of the adolescent and parent perspective. Themes and subthemes were reviewed and revised by regular discussion with other research team members (TR, PW, and CC) to develop the final set of themes/subthemes. During these discussions, the research team also reflected on the lead researcher’s and the whole research group’s prior assumptions and knowledge in the field of help-seeking. Finally, JR produced a report of the analysis by elaborating identified themes and subthemes and using data extracts (quotes) related to the research question.

## Results

We identified four themes that describe barriers/facilitators to seeking and accessing professional help among adolescents with a diagnosis of an anxiety and/or depressive disorder: (1) making sense of difficulties, (2) disclosing problems, (3) ambivalence to seeking professional help, and (4) the instrumental role of others. Barrier and facilitator subthemes identified within each overarching theme, together with exemplary quotes are outlined in Table 3.

1. Making sense of difficulties (‘I just thought I was my kind of normal’)

**Table 3:** Barrier and facilitator subthemes identified within each theme and exemplary quotes

Theme	Barrier and facilitator subthemes	Description	Exemplary quotes (Pseudonym, age, ADIS-C/K-SADS diagnoses)
Making sense of difficulties (‘I just thought I was my kind of normal’)	Recognising anxiety and depression symptoms and knowing where to get help	Adolescents report recognising some anxiety and/or depression symptoms, especially those related to physical sensations and behaviour. In addition, they often ‘classify’ themselves or other people as anxious/depressed based on someone else’s (e.g. their parent’s, friend’s, GP’s) naming of symptoms as anxiety/depression. Adolescents are also often not aware of available help for their problems, apart from the support offered in their schools	‘if they would be breathing really faster they’ll probably have anxiety attack…depression is like when they’ve got a head in their hands or something or they’ve like got a really sad face on their face all the time’. (Savannah, 11, SocA, GAD) ‘when I asked my mum about it, once I asked her if we know anyone who had it (anxiety), mum would say that a lot of my cousins have it’ (Zara, 12, GAD, SocA) ‘I know the student support centre (in school) can help me and he (the school counsellor) can most likely help me…and that’s about it’ (Hannah, 14, PD, SocA, Agor, GAD)
	Beliefs about mental health and help-seeking	Adolescents’ understanding of their difficulties appears to be influenced by their beliefs about mental health and help-seeking, such as a belief that help-seeking is brave and that mental health problems are common and (not) ‘normal’. Adolescents that described themselves as ‘not normal’ compared to other people, especially their peers, tended to be the ones without a prior experience of professional help	‘I’d think they (friends) would be quite brave for doing that (seeking help) and I’d be proud of them for getting help’ (Lilly, 16, GAD, SocA, PD, Agor, MDD, Dyst, SepA) ‘…Not many people that I’ve met personally go through the same things that I am. Like fears and stuff like that. They’re just like, I guess you can say normal’. (Chris, 15, PD, GAD, Agor)
	Taking opportunities to learn about mental health	Adolescents report wanting to have more opportunities to learn about specific mental health problems and available help, however, their engagement in existing opportunities appears to be relatively low, and even if they are provided with the information directly (e.g. given leaflets with information resources), adolescents do not seem to engage fully in these opportunities. Distributing information via popular social media (e.g. Instagram and Snapchat) is suggested as a way of facilitating their engagement	‘…They could make like links on the computers to, to healthcare websites…It would be like the phone numbers, and places you could go to to get healthcare and talk about what you are going through’. (Ben, 12, MDD, GAD) ‘…I’ve not personally used it (Kooth), but I know it’s there’ (Isaac, 13, MDD, PD, SocA, GAD, SpecP) ‘…When it comes to the social media, it would have to be something that is already in that apps that people use now. Because I don’t think anyone would like, there’s always the idea of like ‘oh let’s make a mental health app’, like not many people would actually get that to help themselves’ (Alex, 15, GAD, SocA)
	Differentiating between anxiety/depression symptoms and a person’s attributes	Adolescents, parents and teachers struggle with differentiating between anxiety/depression symptoms and adolescent’s attributes. For instance, some parents and adolescents report always perceiving their child/themselves as shy and not confident, and therefore perceiving their (child’s) difficulties as personality traits and, consequently, not considering help-seeking. Furthermore, parents often attribute adolescents’ behaviour to characteristics of adolescence (e.g. moodiness, constant worry). Adolescents suggest that some school-based interventions (e.g. mental health screening, mental health assemblies) could help themselves and others identify anxiety/depression symptoms that require professional help	‘Well she’s naturally quite a shy child…you know, she’s not a sort of outgoing child, she is naturally quite shy, so I think that’s hold her back a lot’. (Victoria (mother), 11, SocA) ‘I think possibly he’s got lots of things going on with his mind, but I think he’s kind of a typical fifteen year old’ [Chris (mother), 15, PD, GAD, Agor] ‘…Basically doing the whole school (screening). Like, I think it should be like, in the law. Like someone every, maybe two, one or 2 years a person comes in like a teacher in the room and just like we’re part of like counselling thing and if you have any worries, we can help you’ (Tina, 15, GAD, SocA, Dyst)
Disclosing problems (‘I was scared of telling people how I feel’)	Worrying what other people will think	Adolescents are concerned about being negatively evaluated if they disclose their problems to anyone, and that includes formal and informal sources of help. Although this is less commonly expressed by boys, their parents indicate that they also have these concerns. Concerns about being judged by other people were particularly marked among adolescents with self- and/or parent-reported comorbid gender and sexual identity issues	‘I was scared of telling people how I felt. Cos I thought they will judge me and then they’ll think that’s there’s something wrong with me, and stuff like that…’ (Tina, 15, GAD, SocA, Dyst) ‘…I’d be thinking about, maybe they think I’m….I don’t know, they just might think I’m weird’ (Sophie, 16, GAD, SocA, Dyst) ‘I think it’s more about what other, what his friends are gonna think, cos we’re having this discussion at the moment because he, he’s trying to make up his mind whether he’s bisexual, if he’s gay, if he’s straight…so I think it’s more about his image, you know, he doesn’t want to ask for it (help), because it’s gonna make him look bad with his friends’ [Isaac (mother), 13, MDD, PD, SocA, GAD, SpecP]
	Ability to verbalise feelings	Adolescents report that they can struggle to verbalise their feelings and that this then makes it difficult to be able to disclose their problems to other people. Parents describe how this can lead to anger outbursts, particularly among younger adolescents or those with self-/parent-reported comorbid ADHD (traits) and major depressive disorder diagnosis	‘I don’t really like saying much cos I don’t really know what to say’ (Katie, 13, PD, Agor, GAD, SocA) ‘She just finds it hard to express herself because she doesn’t have the mental capacity to explain in the way that other people can understand…she just tends to lash out cause she finds it easier to express it with anger and physicality, rather than with words’. [Claire (father), 12, MDD, GAD]
	Asking for help	Adolescents commonly struggle with initiating a conversation about mental health and asking for help. They are more likely to share their feelings when a parent or a professional initiates the conversation	‘…I didn’t know how to erm, like, ask for the help. Cos, I don’t know, it's just a lot like, I know I can, but can’t just really go up to someone and say ‘hey, can you help me with this?’ (Alex, 15, GAD, SocA) ‘Well, I always thought that, even though people say they’re fine, you should always take like person out of class, all of the people and students, and just like sit with them, and just be like ‘are you sure’, like ‘everything’s okay, you’ve got no worries?’, erm, and stuff like that. Cos I know if that happened, then I, then I could reach out and get help’ (Tina, 15, GAD, SocA, Dyst)
	Ability to trust other people	Being able to trust other people and perceiving other people as trustworthy is a common reason why adolescents (do not) speak about their feelings to anyone. There are differences in which settings or with whom adolescents feel most able to talk about their difficulties without concerns about information being shared with others; for example, whether this is inside or outside the school environment, with friends or teachers/professionals. Inability to trust other people appears to be particularly pertinent among those with past (negative) experience of professionals or negative life experiences (e.g. family violence)	‘I just don’t really trust teachers, I don’t know, cos they (.), they could be like ‘oh we won’t tell anyone’, but then like, really they get talking to someone, talking to other students what people said or something’. (Joe, 16, SocA, GAD) ‘I have spoken to some (friends) about it but not like everybody…(and to) family members…Cos they won’t like tell anyone else I think’ (Luke, 11, SpecP, GAD, SepA, SocA) ‘…I just don’t really have a lot of trust in anyone’ (Anna, 15, GAD, PTSD, SocA)
	Anxiety and depressive symptoms interfere with help-seeking	The very nature of having an anxiety disorder or depression can get in the way of successful help-seeking. In particular, adolescents report struggling to speak to other people due to their shyness/social anxiety, lack of confidence and feelings of hopelessness	‘I just don’t really feel that confident to do that…to speak to anyone I think’. (Lilly, 15, GAD, SocA, PD, Agor, MDD, Dyst, SepA) ‘I carry on and just keep going. Just kind of… do the same thing. Cause nothing is going to change anything’ (Sophie, 16, GAD, SocA, Dyst)
	Concerns about the impact on others	Although adolescents’ friends and families usually represent a first source of help for adolescents, adolescents do not always share their feelings with these people as they do not want them to worry about them or make their parents angry. This barrier is common among older adolescents and also voiced by parents of adolescent boys	‘It’s just, it’s like, it’s almost uncomfortable cos I don’t want them (parents), I don’t want to tell them cos I don’t want, want them to worry about me’. (Isaac, 13, MDD, PD, SocA, GAD, SpecP) ‘…I don’t like talking about myself to them (friends), I’d rather listen to what they have to say and that stops them from worrying about me…I thought that she (mother) was going to be angry with me (if I tell her)’. (Hannah, 13, PD, SocA, Agor, GAD) ‘Ben keeps all for himself. I think it’s because I have girls and he’s the oldest he doesn’t want to put any stress on me’ (Ben (mother), 12, MDD, GAD)
Ambivalence to seeking professional help (‘There’s like a part of me that wants help and a part that doesn’t’)	Desire to be self-reliant	Adolescents report a preference to rely on themselves when facing emotional difficulties, and wanting to show themselves and others that they are strong enough to cope on their own. As such, they can perceive help-seeking to be in conflict with their perceptions of themselves. Parents sometimes explain their children’s self-reliance by referring to their own ways of coping with difficult emotions. Similarly, parents of boys report barriers related to their sons appeared to see help-seeking as conflicting with the idea of what it is to be male	‘…because I think that’s something that I have to do by myself…cos I’m a tough person’ (Chris, 15, PD, GAD, Agor) ‘I think it’s just my pride… cos I think I can do everything by myself.’ (Victoria, 14, SocA) ‘I think cos I’ve got my own problems and I don’t ask for help and I do everything by myself I think she thinks she’s got to be the same’ [Savannah (mother), SocA, GAD]
	Other’s reactions	Adolescents are often concerned about being treated differently or being perceived as wanting attention if they reach out for help. Concerns about other people’s reactions appear to be particularly common among those without prior experience of professional help, older adolescents and adolescent males	‘So, I don’t want people to be like, ‘oh, she wants attention' or like, I just don’t like things to be all about me’ (Sophie, 16, GAD, SocA, Dyst) ‘I just don't want people to treat me differently and like take pity on me, I'd rather them just treat me normally…than just be like ‘oh, he’s depressed, you gotta be careful with him,', so yea’. (Frank, 14, MDD, GAD, SocA)
	Fears and expectations about professional help	Not knowing what to expect from professional help (e.g., what professional help will consist of and how the adolescent will react), and whether this will be helpful, represent notable barriers to help-seeking. Adolescents report wanting more information about what professional help looks like, which could reduce their anxiety and help them decide whether to seek help or not. Past (positive) experiences of professional help can also reduce adolescents’ fears and create more realistic expectations about professional help	‘…he’s got himself quite stressed and anxious about the doctor's appointment, he didn’t sleep the night before that and then he got tearful while we were waiting…and I was trying to explain that they won’t do anything, we're just gonna come and have a chat, they're not gonna remove him or anything..’ [Ben (mother), 12, MDD, GAD] ‘I'm happy and scared, like I'm happy cos I’m getting help but then I'm also scared of what will actually happen’ (Lilly, 15, GAD, SocA, PD, Ago, MDD, Dyst, SepA) ‘…I was scared to get help in Year 6 because I didn’t know what’s going to happen, but it kind of comforted me because I knew it’s going to be, I knew it’s not going to be anything scary…It was just something nice’. (Zara, 12, GAD, SocA)
The instrumental role of others (‘If it wouldn’t be for X, I would still be suffering’)	Recognising the need for professional help and initiating the process of help-seeking	Adolescents’ parents and teachers are crucial in the process of accessing professional help. However, although they might identify symptoms of anxiety/depression in an adolescent, they do not always recognise the need for professional help and/or initiate the process of help-seeking. Parents/professionals who perceive an adolescent's symptoms as risky or severe (e.g. self-harming) and interfering (e.g. child not being able to go to school) are likely to initiate the process of help-seeking for a young person	‘He does sometimes have panic attacks…sometimes he just has to erm take himself out of his lessons, but I think, he’s kind of getting into grips with that’. [Chris (mother), 15, PD, GAD, Agor] ‘My mum ran the doctors to book an appointment, cos she said like, she wants me to get help I need’ (Anna, 15, GAD, PTSD, SocA) ‘I think the school's done it (arranged help) when he first started and he was getting quite distressed in a couple of days, I think they’ve obviously assigned him to her (school nurse) and he’s just carried it on with her’. (Luke (mother), 11, SpecP, GAD, SepA, SocA)
	Knowledge of services	Knowing where to seek help is an important facilitator, and adolescents and their parents indicate that schools are most commonly the first nominated source of help for adolescents and their families. Besides from schools, families also turn to their child's GP to seek support. Parents generally lack knowledge about child and adolescent mental health services, unless they have personal or professional experience of CAMHS	‘…mum called up to school to say like, I don’t want to come into school anymore’ (Tina, 15, GAD, SocA, Dyst) ‘…I actually didn't know there was help for erm the children’. [Frank (mother), 14, MDD, GAD, SocA] ‘…I also work for the NHS Trust so I know a lot of services that are available, cos obviously I work for the NHS company’ (Luke (mother), 11, SpecP, GAD, SepA, SocA)
	Family’s resources and resilience	Adolescents and their parents describe the high demand on local child and adolescent mental health services as preventing access and that persistence is required to successfully access services. Parents with sufficient resources (e.g., emotional and financial resources) appear most likely to be able to access specialist support, and this sometimes means accessing help privately. Where parents lack these resources, they may not attempt to seek help for their child. The role of the school seems to be especially important in families with limited resources	‘Nothing stopped us it’s just erm I mean obviously for a while we were waiting to see if we can get somewhere through the doctor but, so that stopped us for a little while, but then we just said ‘no we gotta deal with it’ and we paid for it once, I mean if people haven’t got money, huh, that leaves them there doesn’t it?’ [Joe (mother), 16, SocA, GAD] ‘I’ve been trying to get hold of them (CAMHS) for quite a while now….but to be honest with you…things were going on at the time, do you know what I mean…I just had so much going on I didn’t know if I was coming or going to be honest with you’ [Maya (mother), 13, Agor, SocA, GAD, Hallucinations and Delusions]
	Caregivers and schools working together	Families vary significantly in their experiences of support from other agencies (e.g. schools, GP) when trying to access help. Parents and adolescents describe the importance of feeling well supported by their child’s school to be able to successfully access professional help	‘so me and my husband went into school and said ‘look, we need help, what do we do’, obviously we didn’t, I wouldn’t know how to deal with something like that and they basically helped us and we helped them, if you know what I mean, so we worked together’ [Isaac (mother), 13, MDD, PD, SocA, GAD, SpecP]
	Young person’s engagement with help-seeking	Adolescents may not feel ready for professional help or engage in the process of help-seeking after this has been initiated by parents/schools (e.g. making an appointment with the GP). In these situations, parents report feeling frustrated and hopeless	‘she absolutely had a meltdown when I said I was going to take her to the doctors, to discuss what’s going on and I think she needs help’. [Katie (mother), 13, PDD, Agor, GAD, SocA] ‘at the same time Alex, he refused to to to talk to anybody at this stage and now if Alex, he refuses he wouldn’t, erm so we are stuck for those year, two years’ time completely stuck’ [Alex (mother), 15, GAD, SocA]

*Agor *agoraphobia, *Dyst *dysthymia, *GAD *generalised anxiety disorder, *MDD *major depressive disorder, *PD *panic disorder, *SocA *social anxiety disorder, *SpecP *specific phobia

Adolescents struggle with recognising anxiety and depressive symptoms, understanding what is normal or not and knowing where to get help for their difficulties. They appear to perceive physical sensations (e.g., rapid breathing) and behaviours (e.g., running away from home) as the main features of anxiety/depression and classify themselves or other people based on someone else’s (e.g., GP, friend, parent) labelling of symptoms as anxiety/depression. Adolescents’ understanding of their difficulties if influenced by their beliefs about mental health and help-seeking, such as perceiving mental health problems as ‘normal’ or not, and those adolescents without prior experience of help-seeking are more likely to see their problems as ‘not normal’. Adolescents, especially those at the upper end of the age range, report wanting more opportunities to learn about the signs and symptoms of anxiety and depression through online resources, social media, and research projects. However, their engagement with existing resources is low, and even when provided with information directly (e.g., through study information leaflet), adolescents report that they do not always independently seek it out. While parents and school staff may be instrumental in helping to identify that a young person has symptoms of anxiety/depression and may need professional help, they also appear to struggle to distinguish between the symptoms of anxiety/depression, their child’s attributes, and characteristics of adolescents in general (e.g., being more worried, shy, and withdrawn). Adolescents suggested interventions that could facilitate the identification of anxiety/depression, including screening for anxiety and depression in schools, regular school assemblies on anxiety and depression, distributing information via social media, and educating teachers and parents on warning signs of anxiety and depression.

2. Disclosing problems (‘I was scared of telling people how I feel’)

Adolescents with anxiety and/or depressive disorders find it hard to disclose their problems to other people, from friends and family to professionals. Feeling embarrassed about their feelings and concern about being negatively evaluated by their peers or by adults due to high levels of shame and stigma associated with mental health problems are often reported by adolescents. Adolescents report that, even if they want to speak to other people about their difficulties, they struggle to verbalise their feelings. Barriers related to difficulties with verbalising their problems were especially pertinent among younger adolescents and adolescents with ADHD and/or major depressive disorder. Adolescents can prefer it if other people (e.g., their parents or professionals) initiate the conversation about mental health. When deciding who to speak to, adolescents need to perceive the person as trustworthy, although the type of help adolescents identify as trustworthy varies considerably (e.g. formal vs. informal, help within vs. outside the school). Barriers related to (lack of) trust seem to be especially common among adolescents with past negative life or experiences (e.g., family violence) or (negative) experience of professional services. Notably, adolescents who feel unable to share their feelings with professionals also describe not feeling able to open up to friends or family either. Some specific anxiety and depressive symptoms, such as shyness, quietness, lack of confidence, and hopelessness, seem to contribute to difficulties disclosing problems to others. Older adolescents and adolescent boys, in particular, can be also worried about making other people, especially their parents and friends, upset if they disclose their problems to them.

3. Ambivalence to seek professional help (‘There’s like a part of me that wants help and a part that doesn’t’)

Adolescents are unsure about whether they want professional help for their difficulties or not. One of the main barriers that stops adolescents from seeking professional help is a preference to rely on themselves, and some parents highlighted that adolescents may have adopted this coping style through observing their parents. Perceived gender roles appear to play a significant role here, with adolescent males being more likely to hold beliefs of needing to be strong and handling things on their own. Furthermore, older adolescents and adolescents without prior experience of professional help seem to be especially concerned about being able to cope with their problems on their own, and feeling ‘too proud’ to reach out for professional help. Adolescents also seem to be concerned with how other people will react if they seek professional help, and adolescents who worry about being perceived as ‘attention seekers’ or ‘weak’ by other people are less likely to seek professional help. Concerns about other people’s reactions seem to be more common among older adolescents, adolescent males, and those without a previous experience of professional help. Finally, adolescents’ fears and expectations about professional help also play a significant role in decisions about whether to seek professional help. Adolescents with past (positive) experience of professional help-seeking are more likely to hold positive expectations, are less afraid of professional help, and more likely to seek professional help in the future than those without these past experiences.

4. The instrumental role of others (‘If it wouldn’t be for X, I would still be suffering’)

Adolescents do not appear to access professional help on their own—they need their parents and/or school staff to arrange professional help for them. If parents and teachers perceive adolescents’ problems as severe (e.g., self-harm) and interfering (e.g., adolescent not being able to attend school), they are more likely to seek and access help. However, parents and schools are not always aware of available help for their child’s anxiety and/or depressive disorders. Families report turning to adolescents’ schools and GPs most commonly, and the role of these professionals in referring families to appropriate help is invaluable. Experiences of help-seeking/accessing among families differ significantly, and the family’s emotional and financial resilience and resources also play an important role in whether a family will access professional help or not. Adolescents and parents report that the school’s level of engagement and support in the process of accessing professional help is important and particularly crucial when parental resources are limited (e.g., when parents are struggling with their own mental health difficulties or in families with a very low socioeconomic status). Finally, even though adults around adolescents usually lead the process of accessing professional help, adolescents themselves may not always feel ready to engage in the help-seeking process, which can be a source of frustration for parents.

## Discussion

This study captured the perspectives of adolescents identified in the community who met diagnostic criteria for anxiety and/or depressive disorders on seeking and accessing professional help for their mental health problems. We identified a complex array of barriers and facilitators that influence adolescents’ decisions about seeking help. The study particularly highlights the instrumental role of adults, especially parents, in enabling adolescents to access professional help successfully.

Barriers and facilitators to seeking and accessing professional help among adolescents with anxiety and/or depressive disorders reflect many of the normative characteristics of the adolescent developmental period. For instance, adolescents report their parents’ and school staff’s difficulties in distinguishing between the symptoms of anxiety/depressive disorders, and behaviours that are perceived as ‘typical’ for this age (e.g., fluctuations in mood, appearing worried, withdrawn or disengaged). To receive support for their emotional difficulties, adolescents need to disclose their problems to other people, and adolescents report struggling to do that (including to friends), mainly due to fears of negative social consequences which are typically heightened in adolescence [37]. The growing need for independence and autonomy that is central to adolescence [38] was also reflected in our findings. Adolescents reported struggling to find a balance between wanting to be independent and the need for other people’s help and support, and commonly relied on adults, particularly their parents and school staff, to access professional help.

Our findings are broadly consistent with previous research [8, 39] and existing help-seeking models for adolescents, such as Rickwood et al.’s model of help-seeking for mental health problems in young people [17]. This model, developed for young people aged 14–24 and for help-seeking for various mental health problems, proposes four stages of help-seeking: (1) awareness of symptoms and appraisal that assistance might be required; (2) expressing of awareness and appraisal in words, so they can be understood by other people; (3) availability of sources of help; and (4) willingness of the adolescent to disclose their difficulties to the selected, available source. Indeed, it appears that each of these barriers may potentially be heightened in (1) people with anxiety/depressive disorders, due to the tendency to avoid anxiety-provoking situations and to procrastinate among participants with diagnoses of anxiety disorders, and the lack of motivation, negative self-perception and hopelessness among participants with depressive disorders, and (2) adolescents who experience particular concerns about negative evaluation from peers, family, and professionals, and are developing a particular need for autonomy.

Our findings have clear practical implications for reducing barriers to access to treatment for anxiety and/or depressive disorders in adolescents. Consistent with the previous research [8–10], interventions to improve the mental health literacy of adolescents as well as their parents, school staff, and GPs are needed to minimise barriers related the identification of anxiety/depression in adolescents. Participants in our study suggested that it would be helpful to have regular screening for common mental health difficulties in schools and a larger number of mental health assemblies through which they could be introduced to the ‘warning signs’ of anxiety/depressive disorders. In addition to screening, adolescents suggested that opportunities for regular, informal conversations about mental health, in particular with their parents, could help minimise barriers related to difficulties with verbalising their feelings. Adolescents also suggested greater availability of online information resources and help, especially through social media. However, previous research [40] has suggested that adolescents’ engagement in online resources is relatively low, and therefore, the ways of accessing online support need to be carefully considered (e.g., through formal settings, such as in schools) [40]. In addition, strategies are needed to normalise mental health problems, such as anxiety and depression, and to reduce stigma associated with mental health problems and help-seeking. In particular, efforts need to normalise mental health problems in broader contexts where high levels of stigma may exist (e.g., gender dysphoria). The findings also highlight that explaining and maintaining confidentiality of information are essential. It will be critical that all resources and means of support are developed in partnership with adolescents to meet their specific needs, such as the growing need for autonomy and independence. Our findings also highlight the importance of supporting the adults around an adolescent, especially their parents and school staff who often arrange help for them. To be able to access services, parents need to be informed about anxiety/depression in adolescents and where and how to access help. Adolescents and parents report turning to schools and GPs first and, therefore, it is important that these professionals know what services and support exist and are able to refer families as appropriate [41]. In addition, mental health services need to be available in accessible, so the families can reach them promptly. Finally, our findings suggest that the role of schools in identifying problems and enabling support for adolescents with anxiety and/or depressive disorders is invaluable in cases where family capacities are limited.

### Strengths and limitations

Strengths of the study include the focus on a sample of adolescents (aged between 11 and 18) who met the diagnostic criteria for anxiety and/or depressive disorder and were identified in a community setting by screening a large (> 1,000) number of adolescents and using standardised diagnostic interviews. To our knowledge, this is the first study that identified adolescents with the diagnosis of an anxiety/depressive disorders in a community setting (not a mental health clinic or service), and included those who had either not sought any professional help for their difficulties or not accessed a specialist service. Furthermore, as one of the participating schools was in a severely deprived area of the UK, the experiences of adolescents who are least likely to access specialist mental health services were likely to have been captured [42]. In addition, we used purposive sampling which resulted in a diverse study sample (e.g., with variability in terms of ethnicity, socioeconomic status, comorbid physical and mental health conditions, and previous help-seeking). Finally, we applied different procedures to ensure the rigour of the study, including data triangulation, member-checking, and reflexivity throughout the processes of data collection, analysis, and interpretation. However, it is important to acknowledge the study’s limitations. As only half of the participants that were invited took part in the diagnostic assessment, barriers experienced by adolescents and families that are hardest to reach (e.g., families where parents do not speak English) may not have been captured. Similarly, only adolescents with high level of self-reported anxiety and/or depressive symptoms were invited to take part in the diagnostic assessment and interview, and therefore, the study may have not captured the experience of young people who also meet the diagnostic criteria for anxiety and/or depressive disorders, but were not identified through screening (‘false negatives’). In addition, the lead researcher’s (JR’s) relationship with families from prior data collection and all the research team’s extensive prior knowledge of adolescent anxiety/depression, treatment, and help-seeking inevitably influenced the interpretation of the data.

## Conclusions

Understanding the beliefs and experiences of seeking and accessing help among adolescents with anxiety and/or depressive disorders are crucial to improve access to support and treatment for these most common mental health difficulties. In particular, the perspectives of adolescents themselves need to be addressed, as adolescents can take a more active role in the process of help-seeking and are developmentally significantly different to preadolescent children. Our study identified many barriers and facilitators at the adolescent individual level, as well as at the level of their family, school, and broader context. Improving knowledge about anxiety and depressive disorders, normalising mental health problems and help-seeking, providing age-appropriate support for adolescents, and supporting adolescents’ parents in the process of accessing help are instrumental in enabling these young people to access professional help successfully.

## Supplementary Information

Below is the link to the electronic supplementary material.

Supplementary file1 (PDF 156 KB)

Supplementary file2 (PDF 142 KB)

Supplementary file3 (PDF 156 KB)

## Data Availability

The research materials can be accessed by contacting the corresponding author.
