# Supplementary material for: Adolescents’ perceived barriers and facilitators to seeking and accessing professional help for anxiety and depressive disorders: a qualitative interview study
Source: Eur Child Adolesc Psychiatry. 2021 Jan 27;31(6):891–907. doi: 10.1007/s00787-020-01707-0 (PMC9209355; doi:10.1007/s00787-020-01707-0)
Supplement: Supplementary file 1 — Supplementary file1 (PDF 156 KB) [file 787_2020_1707_MOESM1_ESM.pdf]

Adolescents' perceived barriers and facilitators to seeking and accessing professional help for anxiety and depressive disorders: ***A qualitative interview study***

European Child and Adolescent Psychiatry

Jerica Radez[1,2], Tessa Reardon[1,3], Cathy Creswell[3], Faith Orchard[4] and Polly Waite[1,3]

[1] School of Psychology and Clinical Language Sciences, University of Reading, Reading, RG6 6AL, United Kingdom

[2] The Oxford Institute of Clinical Psychology Training and Research, University of Oxford, Oxford, OX3 7JX, United Kingdom

[3] Departments of Experimental Psychology and Psychiatry, University of Oxford, Oxford, OX2 6GG, United Kingdom

[4] School of Psychology, University of Sussex, Brighton BN1 9RH, United Kingdom

Correspondence to Dr Tessa Reardon (tessa.reardon@psy.ox.ac.uk)

Electronic Supplementary Material 3: Adolescent- and parent-interview topic guides

Adolescent- and parent-interview topic guides

Adolescent interview topic guide

Knowledge and understanding of anxiety and depression in young people

1. Can you tell me a bit about what you know about anxiety and depression?
   - Probe: How can you tell if someone your age has been experiencing anxiety and/or depression?
   - Probe: Do you know someone with anxiety and/or depression? How do you know they have been experiencing these difficulties?

Personal experience of identifying anxiety and/or depression

1. Last time we met I asked you lots of questions about how you've been feeling recently and you told me about your worries and/or low mood. To what extent do you perceive these feelings to be a problem for you?
   - Probe: What makes you think that this is (not) a problem?

2. Have you talked to other people about these feelings?

(for those who have talked to someone before)
   - Probe: Who did you talk to? Was there any particular reason for you to talk to them?
   - Probe: Did that person (e.g. a parent/friend/partner) notice anything? Or did you tell them how you have been feeling first?

   (for those who haven't talked to anyone before)
   - Probe: Have you considered talking to anyone?
   - Probe: Was there anything particular that stopped you from doing that?

Help-seeking attitudes and knowledge about available help/support

1. Do you think it is helpful for young people to talk to other people about feeling anxious/depressed? Who do you think that young people should talk to?
   - Probe: Do you think that young people should talk to people close to them (like family, partners, (online) friends)? Who do you think they should talk to? Is there any particular reason to talk to them?
   - Probe: Do you think that young people should talk to someone else (like teacher, GP, school nurse, year tutor)? Who do you think they should talk to? Is there any particular reason to talk to them?

2. Can you tell me a bit about what you know about available help/support for young people experiencing anxiety and/or depression?
   - Probe: Where did you find out about these things?
   - Probe: Would you know where to find help for experiencing anxiety and/or depression? Where would you go?

3. Some young people who experience anxiety and/or depression want to get some help with these feelings and talk to a professional about it. What do you think about young people who seek professional help for their mental health problems?
   - Probe: How would you describe a person who seeks help for their mental health problems?

- Probe: Do you know anyone who has sought professional help for the problems with their fears, worrying and mood? What do you think of them?

Help-seeking/accessing experience and barriers/facilitators to help-seeking/accessing

1. Have you at any point in the past sought professional help to help you managing your difficult emotions, such as fears, worries and/or low mood?

(for those who did seek help for problems in the past)

Can you describe that experience to me?

- Probe: Who did you seek help with? Was there any reason for choosing them? How was that help for you?

2. Have you sought any professional help for the (current) problems with your fears, worries and/or low mood?

(for those who did seek help for their anxiety and/or depression)

Who did you speak to?

- Probe: Have you tried to speak to someone else than your close ones about your problems (like teacher, GP, school nurse, year tutor)?

3. Has anything stopped you from seeking help?

(for those who have sought professional help)

- Probe: Has anything or anyone helped you when trying to seek help?

(for those who have not sought professional help)

- Probe: Has anything/anyone else stopped you from seeking help?

4. If you did get support from someone. What do you think that that would look like?
    - Probe: Can you describe to me your expectations of that support?
    - Probe: What would you expect from the relationship between you and the professional?
    - Probe: Would you expect that the professional help would help you with dealing with your problems?

5. Assuming that you would decide to seek help. What kind of person would you find the easiest to talk to?
    - Probe: What kind of person would make you want to talk to them about your anxiety and/or depression?
    - Probe: What kind of person would make you trust them?

(for those who have sought professional help)

- Probe: Was there anything about that person that made you decide to speak to them?

(for those who have not sought professional help)

- Probe: Was there anything about the specific person that made you decide *not* to speak to them?

6. Can you think of anything that may make it easier for young people experiencing anxiety and/or depression to seek help?
    - Probe: What do you think that the school could do to make it easier for young people like you to access help?
    - Probe: What do you think that other people around you (like parents, friends) could do to make it easier for young people like you to access help?

- Probe: Can you think of any other tools (such as IT tools, social media) that could make the process of accessing help for your fears, worries and/or low mood easier?
- Probe: What do you think that a process of help-seeking would look like in the ideal world?

Parent interview topic guide

Knowledge and understanding of anxiety and depression in young people

1. Can you tell me a bit about what you know about anxiety and depression in young people?
- Probe: How can you tell if a young person has been experiencing anxiety and/or depression?
- Probe: Do you know any young person with anxiety and/or depression? How do you know they have been experiencing these difficulties?

Experience of identifying anxiety and/or depression in their child

1. Last time I met with your child I asked them lots of questions about how they've been feeling recently and they told me about their worries and/or low mood and I sent you both a report summarising what they told me. Can you tell me what do you think about that report?
- Probe: Did anything in the report come as surprise?
- Probe: Did you (or anyone else in the family) notice any change in your child? What did you notice?

(for those who have noticed changes in their child)

- Probe: Can you tell me when was the first time you thought your child may be experiencing problems with anxiety and/or depression?
- Probe: What made you think that they are experiencing these difficulties?
- Probe: Have you done anything in particular to understand what's been going on with your child (e.g. spoken to someone, or did a research on anxiety/depression)?

2. Have you and your child ever talked about their worries/low mood? Can you tell me a bit about that?

(for those who have talked with their children before)

- Probe: Do you remember who initiated the conversation?
- Probe: Do you remember what was going on for your child at that time? How long ago was that?
- Probe: Do you remember how the conversation went? Do you think that your child found it helpful?
- Probe: Was there anything that stopped you from having these conversations?

(for those who have not talked with their children before)

- Probe: What do you think that stopped you or made it hard for you to have these conversations?

3. Have you and your child talked recently about their fears, worries and/or low mood?
- Probe: Do you remember who initiated the conversation?
- Probe: Do you remember how the conversation went? Do you think that your child found it helpful?
- Probe: Was there anything that stopped you from having these conversations?
- Probe: Have you and your child spoken about getting support from other people, such as their school nurse, GP, year tutor?

(for those who have not talked with their children recently)

- Probe: What do you think that stopped you from having these conversations?

4. Do you think that your child talked to anyone (else) close to them about difficulties with their fears, worries and mood? Who do you think they talked to? What do you think that made them talk to them?
- Probe: Do you think that your child talked to someone else (like their GP, school nurse, year tutor) about difficulties with their fears, worries and mood? Who do you think they talked to? What do you think that made them talk to them?
- Probe: Have you spoken to any of those people on your child's behalf/about your child's difficulties?

(for those who have not talked to anyone else)
- Probe: What do you think that stopped you and/or your child from having these conversations with other people?

Help-seeking attitudes and knowledge about available help/support

1. Do you think it is helpful for young people to talk to other people about feeling anxious/depressed? Who do you think that young people should talk to?
   - Probe: Do you think that young people should talk to people close to them (like family, partners, and (online) friends)? Who do you think they should talk to? Is there any particular reason to talk to them?
   - Probe: Do you think that young people should talk to someone else (like teacher, GP, school nurse, year tutor)? Who do you think they should talk to? Is there any particular reason to talk to them?
   - Probe: Do you think it is helpful for parents to talk to other people if their child is feeling anxious or low?

2. Can you tell me a bit about what you know about available help/support for young people experiencing anxiety and/or depression?
   - Probe: Where did you find out about these things?
   - Probe: Would you know where to find help if your child would be experiencing difficulties with anxiety and/or depression? Where would you go?

Help-seeking/accessing experience and barriers/facilitators to help-seeking/accessing

1. Have you at any point in the past tried to get professional help to help you and your child managing their difficult emotions, such as fears, worries and/or low mood?

(for those who did seek help for problems in the past)

Can you describe that experience to me?
- Probe: Who did you seek help with? Was there any reason for choosing them? How was that help for you?

(for those who did not seek help for problems in the past)

How do you think that the help would look like?

2. Have you sought any professional help for the (current) problems with your child's fears, worries and/or low mood?

(for those who did seek help for their anxiety and/or depression)

Who did you speak to?
- Probe: Have you tried to speak to any professional (like your child's GP, year tutor, school nurse)?

3. Has anything stopped you from seeking help for your child's fears, worries and/or low mood?

(for those who have sought professional help)

- Probe: Has anything or anyone helped you when trying to seek help?

(for those who have not sought professional help)

- Probe: Has anything/anyone else stopped you from seeking help?

4. Can you think of anything that may make it easier for young people experiencing anxiety and/or depression and their parents/families to seek help?

- Probe: What do you think that the school(s) could do to make it easier for young people like you to access help?
- Probe: What do you think that close to young people (such as you – parents, and friends) could do to make it easier for young people like you to access help?
- Probe: Can you think of any other tools (such as IT tools, social media) that could make the process of accessing help for young people's fears, worries and/or low mood easier?
- Probe: How do you think that a process of help-seeking would look like in the ideal world?
