# Supplementary material for: Adolescents’ perceived barriers and facilitators to seeking and accessing professional help for anxiety and depressive disorders: a qualitative interview study
Source: Eur Child Adolesc Psychiatry. 2021 Jan 27;31(6):891–907. doi: 10.1007/s00787-020-01707-0 (PMC9209355; doi:10.1007/s00787-020-01707-0)
Supplement: Supplementary file 2 — Supplementary file2 (PDF 142 KB) [file 787_2020_1707_MOESM2_ESM.pdf]

Adolescents' perceived barriers and facilitators to seeking and accessing professional help for anxiety and depressive disorders: *A qualitative interview study*

European Child and Adolescent Psychiatry

Jerica Radez[1,2], Tessa Reardon[1,3], Cathy Creswell[3], Faith Orchard[4] and Polly Waite[1,3]

[1] School of Psychology and Clinical Language Sciences, University of Reading, Reading, RG6 6AL, United Kingdom

[2] The Oxford Institute of Clinical Psychology Training and Research, University of Oxford, Oxford, OX3 7JX, United Kingdom

[3] Departments of Experimental Psychology and Psychiatry, University of Oxford, Oxford, OX2 6GG, United Kingdom

[4] School of Psychology, University of Sussex, Brighton BN1 9RH, United Kingdom

Correspondence to Dr Tessa Reardon (tessa.reardon@psy.ox.ac.uk)

Electronic Supplementary Material 2: Detailed description of the process of recruitment

Detailed description of the process of recruitment

Obtaining ethics approval: The ethics application for the current study was submitted to the University of Reading Research Ethics Committee (UREC) in June 2018. At the time, changes to the data protection law (GDPR) were introduced, which required the usage of active consent (i.e., opt-in) to process personal data. As there are many limitations to using an opt-in approach in the field of mental health (e.g. systematic exclusion of certain clinical and demographic groups of participants), a special case outlining the benefits of using the opt-out procedure in the current study was made to the UREC. In addition, the study met the GDPR criteria for using opt-out procedure by 1) 'carrying out the task in public interest', and 2) processing personal data 'transparently' (i.e., including detailed information leaflets and privacy notices). The study was, therefore, granted ethics approval immediately (in August 2018).

Recruiting schools: Thirty-one state secondary schools in Berkshire, Buckinghamshire, London, North England and Oxfordshire were approached from September to November 2018. Invited schools had no academic entry criteria and were not taking part in other University research projects. Three large mixed state schools (all in Berkshire) agreed to take part, and one school withdrew prior to the data collection.

Screening for high levels of symptoms of anxiety and depression: Participating schools distributed information leaflets and opt-out consent forms to 1,706 parents/carers. After two weeks, the lead researcher (JR) administered paper forms of the adolescent-report questionnaires with students aged 11-18 (Year 7-Year 13), whose parents/carers had not opted out (97.7%). Of 1,237 students invited to take part, 94.2% provided consent/assent and completed the questionnaires (see Appendix C). Adolescent background information (gender, ethnicity, date of birth, school year, whether an adolescent is a learner of English as an additional language, whether an adolescent is eligible for free school meals and whether an adolescent has a statement for special educational needs) was collected from school records at the same time. Where questionnaire responses indicated any risk or raised concerns about the young person's safety, this was reported to the schools' safeguarding teams immediately.

Identifying participants for diagnostic assessments (ADIS-C and K-SADS): Of 1,165 collected questionnaire responses, 417 (35.8%) adolescents had at least one RCADS-C/MFQ-C subscale/scale

score above the clinical threshold (RCADS-C subscale *T*-score > 70 and/or MFQ-C total > 26). To avoid false positive identification and due to lack of resources do administer 417 diagnostic assessment, the threshold to identify the pool of participants for the diagnostic assessments was set to at least 4 (out of 8) RCADS-C/MFQ-C subscale total scores within the 'clinically elevated' range (n = 121). We then purposively sampled from this pool of participants to ensure that adolescents invited to the diagnostic assessment varied on the following characteristics: age, gender, ethnicity, prior help-seeking/accessing experience, family's socioeconomic status, and presence/absence of a statement of special educational needs. Fifty-two adolescents were identified, and invited to take part in the diagnostic assessment. Parent/carer consent forms, consent forms for audio recording the assessment, and the information leaflet outlining the purpose of the assessment were distributed to families electronically. Up to three follow-up phone calls were made to each family within two weeks of sending an initial invitation to the assessment. After parents/carers provided consent, the lead researcher (JR) administered the assessment one-to-one with adolescents only in a quiet room in the adolescent's school. Prior to the assessment, each adolescent also provided assent/consent to take part in the assessment and for the researcher to audio record the assessment. If any adolescent became upset during the interview, they were encouraged to take a break or reminded they could withdraw from the study completely. Where an adolescent's responses indicated any risk or raised concerns about the young person's safety, this was reported to the school's safeguarding team immediately. Each adolescent who took part in the assessment was given a £10 voucher to reimburse them for their time.

Identifying participants for qualitative interviews: Following the diagnostic assessment, each family received a detailed report outlining the 1) outcome of the assessment and 2) recommendations for further treatment. If appropriate, families were offered support for their child's anxiety and/or depressive disorder within the university-based research clinic. Of 26 adolescents who took part in the diagnostic assessment, 24 met the DSM-5 diagnostic criteria for a current anxiety and/or depressive disorder. Approximately two weeks after the assessment, families were re-contacted by JR over the phone and invited to take part in the qualitative interview study. Adolescents, as well as their parents/carers, were invited to take part at this stage, and the lead researcher explained the purpose of the qualitative interviews. Parents/carers were asked to provide consent for an interview with them,

their child, and for audio recording both interviews. Each family was contacted up to three times to provide consent for the qualitative study. Of 24 families invited, 22 provided consent for the adolescent and the parent/carer interview.

Interviews were firstly conducted with each adolescent and a parent interview was scheduled within one week after the interview with their child and was partially guided by things reported in adolescent interview. As with the diagnostic assessments, all qualitative interviews with adolescents were conducted one-to-one in their school, and any risk concerns were reported to the schools' safeguarding teams immediately. Prior to the qualitative interview, each adolescent was asked to provide assent/consent to take part in the interview and to be audio recorded. Adolescents' parents/carers were interviewed at a time convenient for them and over the phone. Among the 22 families who provided consent to take part in the qualitative study, all 22 adolescents and 20 parents/carers took part. Two parents/carers did not take part without giving any reason. Each family that took part in the qualitative interview was given £10 voucher to reimburse them for their time.
