# Supplementary material for: Adolescents’ perceived barriers and facilitators to seeking and accessing professional help for anxiety and depressive disorders: a qualitative interview study
Source: Eur Child Adolesc Psychiatry. 2021 Jan 27;31(6):891–907. doi: 10.1007/s00787-020-01707-0 (PMC9209355; doi:10.1007/s00787-020-01707-0)
Supplement: Supplementary file 3 — Supplementary file3 (PDF 156 KB) [file 787_2020_1707_MOESM3_ESM.pdf]

Adolescents' perceived barriers and facilitators to seeking and accessing professional help for anxiety and depressive disorders: ***A qualitative interview study***

European Child and Adolescent Psychiatry

Jerica Radez[1,2], Tessa Reardon[1,3], Cathy Creswell[3], Faith Orchard[4] and Polly Waite[1,3]

[1] School of Psychology and Clinical Language Sciences, University of Reading, Reading, RG6 6AL, United Kingdom

[2] The Oxford Institute of Clinical Psychology Training and Research, University of Oxford, Oxford, OX3 7JX, United Kingdom

[3] Departments of Experimental Psychology and Psychiatry, University of Oxford, Oxford, OX2 6GG, United Kingdom

[4] School of Psychology, University of Sussex, Brighton BN1 9RH, United Kingdom

Correspondence to Dr Tessa Reardon (tessa.reardon@psy.ox.ac.uk)

Electronic Supplementary Material 1: COREQ Checklist

COREQ Checklist

Topic	Item No.	Guide Questions/Description	Reported on Page No.
**Domain 1: Research team and reflexivity**			
Personal characteristics			
Interviewer/facilitator	1	Which author/s conducted the interview or focus group?	8
Credentials	2	What were the researcher's credentials? E.g. PhD, MD	8
Occupation	3	What was their occupation at the time of the study?	8
Gender	4	Was the researcher male or female?	8
Experience and training	5	What experience or training did the researcher have?	8
Relationship with participants			
Relationship established	6	Was a relationship established prior to study commencement?	8
Participant knowledge of the interviewer	7	What did the participants know about the researcher? e.g. personal goals, reasons for doing the research	Figure 1, ESM 2
Interviewer characteristics	8	What characteristics were reported about the interviewer/facilitator? e.g. Bias, assumptions, reasons and interests in the research topic	8
**Domain 2: Study design**			
Theoretical framework			
Methodological orientation and Theory	9	What methodological orientation was stated to underpin the study? e.g. grounded theory, discourse analysis, ethnography, phenomenology, content analysis	8, 9
Participant selection			
Sampling	10	How were participants selected? e.g. purposive, convenience, consecutive, snowball	Figure 1, ESM 2
Method of approach	11	How were participants approached? e.g. face-to-face, telephone, mail, email	Figure 1, ESM 2
Sample size	12	How many participants were in the study?	5, Table 1, ESM 2
Non-participation	13	How many people refused to participate or dropped out? Reasons?	6
Setting			
Setting of data collection	14	Where was the data collected? e.g. home, clinic, workplace	5
Presence of non-participants	15	Was anyone else present besides the participants and researchers?	5
Description of sample	16	What are the important characteristics of the sample? e.g. demographic data, date	Table 1
Data collection			
Interview guide	17	Were questions, prompts, guides provided by the authors? Was it pilot tested?	8
Repeat interviews	18	Were repeat interviews carried out? If yes, how many?	5
Audio/visual recording	19	Did the research use audio or visual recording to collect the data?	5
Field notes	20	Were field notes made during and/or after the interview or focus group?	8
Duration	21	What was the duration of the inter views or focus group?	8
Data saturation	22	Was data saturation discussed?	9
Transcripts returned	23	Were transcripts returned to participants for comment and/or	N/A

Topic	Item No.	Guide Questions/Description	Reported on Page No.
		correction?	
**Domain 3: analysis and findings**			
Data analysis			
Number of data coders	24	How many data coders coded the data?	9
Description of the coding tree	25	Did authors provide a description of the coding tree?	9
Derivation of themes	26	Were themes identified in advance or derived from the data?	9
Software	27	What software, if applicable, was used to manage the data?	9
Participant checking	28	Did participants provide feedback on the findings?	8
Reporting			
Quotations presented	29	Were participant quotations presented to illustrate the themes/findings? Was each quotation identified? e.g. participant number	Table 3
Data and findings consistent	30	Was there consistency between the data presented and the findings?	10-15
Clarity of major themes	31	Were major themes clearly presented in the findings?	10-12, Table 3
Clarity of minor themes	32	Is there a description of diverse cases or discussion of minor themes?	14-15, Table 3
